# Cross-cultural adaptation of the Job Insecurity Scale (JIS) in Brazil and cross-national analysis of Job Insecurity effects in Brazil and Spain

**DOI:** 10.1186/s40359-023-01156-9

**Published:** 2023-04-14

**Authors:** José Antonio Llosa, Esteban Agulló-Tomás, Sara Menéndez-Espina, Camila Teixeira Heleno, Livia de Olivera Borges

**Affiliations:** 1 Padre Ossó Faculty, University of Oviedo, Oviedo, Spain; 2 Faculty of Psychology, University of Oviedo, Plaza de Feijoo, 33003 Oviedo, Asturias Spain; 3 Universidad Federal de los Valles de Jequitinhonha y Mucuri (UFVJM), Teófilo Otoni, Brazil; 4 Universidad Federal de Minas Gerais (UFMG), Belo Horizonte, Brazil

**Keywords:** Job insecurity scale, Affective job insecurity, Cognitive job insecurity, Validation, Mental health, Precarious work, Cross-national comparative

## Abstract

**Background:**

Job flexibilisation has increased interest in job insecurity and its consequences. Job insecurity, understood as a fear of losing employment, is linked to a deterioration of mental health, social relations or job satisfaction. Its study has been developed primarily in Europe, in the absence of validated psychometric scales in the Latin American context. To bridge this knowledge gap, the aim of this study is to cross-culturally adapt the Job Insecurity Scale (JIS) in Brazil, and secondly, to establish a cross-national analysis between people employed in Brazil and Spain.

**Methods:**

As criteria for the sample, people with formally established employment in Brazil and Spain were selected. For the scale adaptation process, a sequence of EFA, CFA and validity tests are carried out, as well as a multigroup invariance according to the gender variable. The cross-national comparison compares the effect sizes of affective and cognitive job insecurity on the mental health variable measured with the GHQ-28 scale in both countries.

**Results:**

1165 employed people participate in the study, of whom 573 reside in Brazil and 592 in Spain. The results of the scale adaptation show that the JIS is suitable for use in the Brazilian employment context. The scale offers a factorialisation in two dimensions (affective and cognitive) (CFI = 0.993; TLI = 0.987; RMSEA = 0.04; SRMR = 0.049; GFI = 0.999; NFI = 0.980) with good reliability (ω > 0.84). The cross-national comparison shows that job insecurity has a greater weight in explaining the mental health of the employed population in Brazil than in Spain, which is related to higher indicators of job insecurity in the Brazilian context.

**Conclusions:**

With this validation we now have a validated scale of job insecurity validated for the Brazilian context. The comparison between countries shows the need to establish these analyses, since the behaviour of the phenomenon is different in the contexts studied.

## Background

A defining characteristic of today's labour market is its instability, which is recognised by organisations such as the International Labor Organization (ILO) [1]. Beck [2] states that with the advent of globalisation, labour relations are mobile and discontinuous, along the same lines as the ILO [1]. This has led to changes in identity, on the one hand, and in the capacity to generate life projects, on the other [3]. Welfare states are still deeply conceptualised around the idea of stable work, on which the condition of citizenship and ultimately quality of life depend. However, the current labour model has little to do with Keynesian stability logics for employment [4]. In the face of the so-called flexible or flexicurity labour market, there is a need for indicators to study this flexibility and its effects. This is one of the reasons why subjective job insecurity is growing in relevance in the scientific literature [5]. The development of the Brazilian labour context offers a differential evolution with respect to the European context. The deregulation and flexible labour typical of the neoliberal framework took root in Brazil without there having been a full development of the welfare state beforehand [6]. This is due to the country's colonial history and an economy closely linked to the primary sector. All this together with periods of persecution of trade union forces [7]. As a result, the country still has a very large underground economy, which in the neoliberal context is combined with the experience of labour insecurity in labour relations [8].

The concept of job insecurity emerged linked to the stress theories of Lazarus and Folkman [9] and was defined for the first time in the 1980s [10]. Job insecurity is first understood as: "Perceived powerlessness to maintain desired continuity in a threatened job situation" [10]. In this paper, job insecurity is understood as a variable that, among others, explains precarious employment [11]. The first conceptual discussion between objective and subjective job insecurity soon arises [12]. Objective job insecurity responds to objectively identifiable features in employment relations—such as the formal status of the employment contract. Subjective job insecurity focuses on the expectation or experience of insecurity. The flexicurity labour model described above is characterised by a working environment in which mobility is a structural part, whereby it has been observed that stable working conditions are not necessarily perceived as a guarantee of stability [13, 14]. In particular, this phenomenon is accentuated in those workers with lower incomes or whose jobs are affected by some indicator of job insecurity (e.g. temporary or part-time employment) [15]. While the objective conditions of job insecurity, and precarious employment in general terms, are widely studied, the analysis of the experience of this precariousness is not as widespread. For this reason, and It is in this context, is in which that subjective job insecurity gains relevance as a concept for the analysis of working conditions [16]. Looking at the main definitions, subjective job insecurity can be defined as a process of anticipation, involuntary, uncontrollable and related to an employment situation that one wishes to maintain [17–19]. As can be seen, the proposed understanding of the phenomenon of job insecurity is clearly psychosocial [20]. The first approaches, however, focused their analysis on motivational aspects of an individual nature [21, 22]. In contrast, it is worth considering the relevance of the perspective of Lastad [23], which incorporates the idea of the climate of job insecurity. This makes it possible to understand job insecurity as a relational process within the framework of the organisation, enabling an analysis from a psycho-sociological perspective. Recent studies have shown, for example, variations in the experience of job insecurity depending on the general context of the economic crisis [24] or health crisis with COVID19 [25].

Subjective job insecurity can thus be understood as a phenomenon that allows for a psychosocial perspective (interactive analysis between individuals, social relations and contexts of social interaction), and generates a relational and individual impact [20]. Job insecurity has been found to be related to aspects of personal health and well-being, both mental [15] and physical [26]. With family relationships [27, 28]. Also with job attitudes, such as commitment [29] or the intention to change jobs [29, 30]; as well as interpersonal behaviour in the workplace, such as workplace harassment [31] or lack of collaboration at work [32, 33], and so on and so forth. Two consequences of job insecurity should be emphasised here: general mental health and job satisfaction. Deterioration of mental health has been found to be linked to high job insecurity scores [15, 34]. In terms of job satisfaction, the relationship was inverse: the higher the job insecurity, the lower the job satisfaction [16, 35].

Another important dimension of perceived job insecurity is that which refers to gender. Authors such as Shoss point out the relevance of studying possible gender differences, since differential behavior between men and women was found [20]. Studies revealed that men tend to experience higher rates of perceived job insecurity [36]. It was shown that, even when these differences do not exist, the phenomenon of job insecurity is explained on the basis of different variables for men and women [37, 38]. In the case of men, job insecurity has been explained in terms of variables related to career development, and the possibility of losing their job is a professional obstacle. In the case of women, job insecurity was more closely related to conditions of precariousness. For example, a limitation in maintaining living conditions in the face of the possibility that the job may disappear.

### Measurement and dimensions of Job Insecurity

The relevance of job insecurity as a concept of analysis in Work and Organisational Psychology is reflected in the increase in scientific publications related to it [15]. This interest has been accompanied by the creation of several measurement scales, which have helped in its conceptualisation and dimensioning [36]. Although a measurement of job insecurity has sometimes been proposed with just a single item [37–39], meta-analytic analysis comparing single-item measures and psychometric scales shows that single-item measurements are not adequate to understand the conceptual complexity we face [40]. When dealing with psychometric scales, the first instrument that approaches the measurement of job insecurity is by Caplan et al. [41] with a one-dimensional measurement. Job insecurity is also included in the scale of Jonshon et al. [42] as one of the 5 dimensions of the Work Opinion Questionnaire. Again, the approach is one-dimensional. There are now highly reliable scales for measuring subjective job insecurity as a unitary construct, such as the validation in five European countries of the test developed by Vander Elst et al. [19] or the recently validated 4-item QUAL-JIS scale in Romania and Belgium [43]. The concept continues to develop under this perspective, and appraisals of Job Insecurity have recently begun to be assessed with the JIAS-6 test [44]. However, since the work of Ashford et al. [45], the affective and cognitive dimension of the phenomenon began to be measured, gaining relevance with the psychometric proposal by Hellgren et al. [46]. This milestone is important for our study, as it assumes that the concept of subjective job insecurity is not one-dimensional, but is composed of a cognitive and an affective dimension [47]. Cognitive and affective job insecurity both refers to the fear of losing a job that one wishes to keep. However, the understanding of this expectation of loss has been conceptually explained in affective and cognitive terms [48]. We understand the cognitive elements as those related to the belief that the job will be lost, while the affective dimension relates to the emotional reaction of fear to the loss of the job: worry, fear or anxiety about the possible loss [49]. While Sverke et al. [50] in their meta-analysis raise the possibility that affective job insecurity may be the one that most accurately contains the phenomenon that they are trying to measure, the fact is that the literature evidences this dimensionalisation.

This conceptual proposal has made it possible to deduce, through meta-analytical procedures [48] that affective job insecurity is more related to the consequences of job insecurity -whether in relation to job well-being or mental health- than cognitive job insecurity. Secondly, that the two concepts should be treated as distinct. Thirdly, and most importantly, that affective job insecurity functions, in most cases, as a mediator of cognitive job insecurity. That is, affective job insecurity helps to understand the effects of cognitive job insecurity on the job and personal well-being of the employed population. Similar results to these were previously found, showing that the effects of cognitive job insecurity on job satisfaction, organisational commitment and physical well-being were partially mediated by affective job insecurity [49]. Both Jiang et al. [51] and Huang et al. [49] agree on advocating for a dimensionalisation of the construct into affective and cognitive job insecurity. This allows for greater explanatory power for the phenomenon being explored. For this reason, the scale validated in this study on the Brazilian population -Job Insecurity Scale- is the multidimensional job insecurity scale developed by Pienaar et al. [52]. This test has its origin in the scale developed in Dutch with 11 items [53]. It has seen validations in very different contexts, and with different numbers of items. The test by Vander Elst et al. [19] reduces it to 5 items, and its adaptation takes place in five European countries. In the validation proposed in our study, we start from the version of Pienaar et al. [52], with 8 items, originally developed in South Africa, as it is considered the clearest measure of job insecurity in the affective and cognitive dimensionalisation. Its factorialisation is maintained in its adaptation to the Spanish context in 2017 [54]. However, for the time being, no measure of perceived job insecurity among the Brazilian population is available, which is why the main objective of this study is to validate the test in this labour context. This will make it possible not only to have the first validated measure of perceived job insecurity in Brazil, but also to establish comparative relationships with other countries. Hence, the second objective of the paper is a comparative analysis of job insecurity between Spain and Brazil.

### Brazilian and Spanish social and occupational context

When analysing the Brazilian and Spanish social and occupational context in comparative terms, it is worth noting that the labour market in both countries shows important features of precarious work [55, 56]. According to World Bank data for the year 2021, the most recent available, the unemployment rate in both countries is similar: 14.4% in Brazil and 14.7% in Spain [57]. However, with respect to employment conditions, a much higher vulnerable employment rate is observed in the Brazilian labour market in 2019 (28% compared to 11% in Spain) [58]. Other indicators of precariousness, such as part-time employment, also show higher figures in Brazil (14.2% and in Spain 13%) according to OECD data for 2021 [59].

These employment indicators require a broad view of the social situation in each context. Brazil's GINI inequality index stands at 53.5 points, higher than Spain's (34.3 points in 2019) [60].The World Bank poverty indicators also reflect that 5.4% of the Brazilian population lived on less than $1.90 a day in 2019, while it was 0.8% of the Spanish population [61]. This set of data makes it possible to characterise two social and occupational realities on which comparative studies can be established.

### This study

This study presents, as we have already pointed out, two objectives. The first is the cross-cultural psychometric adaptation of the Job Insecurity Scale with the Brazilian working population, and its factorial comparison with the validation carried out in Spain. This involves an analysis of the reliability and validity of the scale, as well as the study of the items and the factorial structure of the scale to determine its dimensionalization [65]. The second objective is a cross-national comparative analysis of the influence of job insecurity on mental health in people employed in Spain and Brazil. This will allow us to explore the behaviour of the construct in both countries.

In the case of the validation process, the constructs of mental health and job satisfaction have been determined as validity criteria. In the case of the Spain-Brazil comparison, the mental health measure was used specifically. These were the variables chosen, since the literature shows a deterioration in job satisfaction determined by job insecurity [66]. The case of mental health is one of the most explored in the scientific literature showing that the greater the job insecurity, the worse the state of mental health [18, 43, 67].

## Methods

### Participants

The sample was composed of 1165 people resident and employed in Brazil (49.18%, n = 573) and Spain (50.82% = 592). The process of data collection was through a non-probabilistic and accidental method. This sampling method involves a non-probabilistic selection of participants according to the criteria for inclusion in the study and the possibility of accessing the sample. It is a type of sampling used when analysing phenomena that are not directly observable, as is the case, and whose incidence in population terms is unknown [69, 70]. We used self-administered questionnaires in online format, through the SurveyMonkey platform. The inclusion criterion for participation in the study was to maintain an active employment relationship at the time of participation in the study. This employment relationship also had to be formally established. This is particularly important in Brazil, where there is a high proportion of informal work [62].

### Instruments

The following scales were administered to the participants in this study in the following order. In the case of the Brazilian sample, the questionnaire used included all four sections to be described: Job Insecurity Scale (JIS) for the measure of job insecurity; Goldberg General Health Questionnaire (GHQ-28) for the mental health screening; European Working Conditions Surveys (EWCS 2010) for the study of job satisfaction, and a final block of questions on socio-demographic aspects. In the case of the Spanish sample, the job satisfaction items were not applied, as they were not necessary for the purpose of the analysis foreseen for the cross-country comparison.

*Job Insecurity Scale* (JIS): The original version of this test for measuring job insecurity was developed by Pienaar et al. [52], originally validated in a South African working population. It consists of 8 Likert-5 items, factorialised into two dimensions: the cognitive dimension is measured in the first four items (α = 0.80) and the affective dimension in the next four items (α = 0.84). The coding of the responses to the items is done with values from 1 to 5; the higher the value, the higher the insecurity score. Items 1 to 5, relating to the cognitive dimension, are reverse coded: score 5 has to be recoded as 1, and so on with the rest of the response levels. This procedure of recoding items from 1 to 5 was also applied in the Spanish version of the test, and was the process followed for this validation study in Brazil. In the Brazilian sample, this is the scale that underwent cross-cultural adaptation, administering the 8 items translated by means of the blind-back method that will be explained in the procedure. In the Spanish sample, the instrument has already been validated [54] with the same number of items and the same structure as in the original scale [52]: a total score (α = 0.88) and two subscales. The first four items are factorialised in the cognitive dimension (α = 0.90) and the following items in the affective dimension (α = 0.78). Three scores can be extracted from this test, a total score and another for each of the dimensions of which it is composed.

*Goldberg General Health Questionnaire* (GHQ-28): with the aim of replicating the original validation of the JIS [52], as well as the Spanish one [54], the measurement of general mental health is administered through this scale originally developed by Goldberg [62]. This psychometric scale presents Likert-4 items. Among the different possibilities of coding the scores, the most common one has been used, which involves assigning values from 0 to 3 to the answers to each item. A higher score on the test implies a worse mental health status. In the Brazilian sample, the adapted instrument was applied [63]. In this case composed of 25 items, and offering a total score in mental health (α = 0.92) and factorialised in three dimensions: somatic symptoms and anxiety (α = 0.89); social dysfunction (α = 0.80), and depression (α = 0.87). For the Spanish sample, the instrument has also been psychometrically adapted [64], in this case maintaining the 28 items of the original, factorialised into four subscales: somatic symptoms, anxiety, social dysfunction and depression. All subscales and scores have α > 0.90 in their adaptation with the Spanish sample.

*European Working Conditions Survey (EWCS 2010),* Eurofound: For the analysis of job satisfaction, the set of 16 items of the Working Conditions Survey 2010 collected in the Eurofound statistical set was applied. These are Likert-5 items. In the Brazilian sample it had McDonald's ω reliability of 0.81. This questionnaire is applied by replicating the process followed in the Spanish adaptation of the Job Insecurity Scale [54]

Socio-demographic data: the final part of the questionnaire used is a set of ad hoc items aimed at collecting socio-demographic information (gender, age, nationality, place of residence and employment status). These items are taken from Eurofound's European Union Labour Force Survey (EU LFS) statistical methodology to ensure that the wording and presentation is checked.

### Procedure

The self-administered questionnaire set was completed in the following order: GHQ-28, EWCS, JIS and socio-demographic data. All participants in the study took part in it voluntarily, giving their consent after being informed of the objectives and data processing of this research. This work was approved by the Ethical Committee of the Psychology Department of the University of Oviedo (Spain), and the Ethical Committee of the Universidade Federal de Minas Gerais (Brazil). In addition, it followed the guidelines set by the Declaration of Helsinki of the World Medical Association (WMA) regarding research work involving contact with people.

In order to respond to the research objectives, two studies were planned. Study 1 with the aim of adapting the JIS-8 subjective job insecurity scale to the Brazilian population; Study 2 established a comparison of the effects of subjective job insecurity on mental health in Brazilian and Spanish workers.

#### Study 1: Job Insecurity Scale (JIS-8), adaptation to Brazilian context

The validation adaptation process followed the phases of the International Test Commission [65]. It posits that adaptation “refers to moving a test from one language and culture to another” [65, p.6], which may involve some modification of the test. Translation is one of the stages of adaptation. However, this process involves studying whether the adapted test maintains the psychometric properties of the original and studying whether both scales measure the same construct [65, 66]. To do so, we followed the sequence of steps proposed by Muñiz et al. [67]: (1) blind-back translation adapting the items to the cultural context under study; (2) analysis of the items and the structure of the test through exploratory factor analysis for the first random half of the sample; (3) confirmatory for the second half, (4) and analysis of the reliability and validity of the test.

Before administering the JIS scale in the Brazilian population, a blind-back translation was carried out [65] to adapt the items to the Brazilian Portuguese language and cultural context. The original version of the instrument was used as a starting point [52], with a first approximation to the translation by the research team to study the appropriate use of its technical language. Subsequently, a group of two independent bilingual people translated the scale from Spanish into Brazilian Portuguese, and another two different people carried out a reverse translation. All versions generated in this process were compared to arrive at the final version of the administered questionnaire.

#### Study 2: Cross-national comparison: Brazil and Spain

For the comparative analysis between Spain and Brazil, the set was administered with the same questionnaires validated in both countries by the research team, achieving comparable results for the analyses.

### Data analysis

The data analysis section was organized under three headings. The first one presented the analysis of the conditions of the sample used; the second one the study of the cross-cultural adaptation of the JIS scale of subjective job insecurity, and the third one the comparative analyses between Spain and Brazil on the relationship between job insecurity and mental health conditions.

#### Sample analysis

First, a descriptive analysis of the sample was carried out. Comparison tests between groups were also established to analyze the socio-labor conditions of the sample from both countries. Chi-square tests (*p* < 0.01) were used to compare the variables gender and type of contract between the Spanish and Brazilian sample, as well as Student's T test (*p* < 0.01) for the age variable.

#### Study 1: Job Insecurity Scale (JIS-8), adaptation to Brazilian context

The exploratory factor analysis was carried out with FACTOR v. 12.01.02 software and applied on a random 50% of the study sample in Brazil. It was developed with a Parallel analysis through 500 bootstrap samples based on polychoric correlations and with a robust ULS extraction method. The EFA based on polychoric correlations is suitable for Likert-5 type items, as in this case [66]. The ULS extraction method is considered optimal in cases where it assumes that the factored scale has a low number of factors and the variables are Likert-type [67, 68]

The CFA, developed with JASP 0.16.3 software and on the other half of the Brazilian sample of the study, estimated the fit through the DWLS method, which is adequate in samples n > 200 [69]. This analysis studied first and second order factors in the factorialisation of the test, using as goodness-of-fit indices: CFI, TLI, RMSEA, SRMR, GFI and NFI. As criterion values for these indices we considered CFI ≥ 0.95; TLI ≥ 0.95; RMSEA ≤ 0.10; SRMR ≤ 0.08; GFI ≥ 0.95; NFI ≥ 0.95 [68, 70]. Also a one-factor with a method factor model to control the effect of reverse code items [71].

In the third instance, in order to test the fit of the scale according to gender (men and women), an invariance test was applied with a restrictive progression analysis sequence. Starting with the configural model, followed by the metric, scalar and, finally, strict [72]. As a criterion for the invariance analysis it was assumed that invariance did not occur when the variance is *Δ* ≤ 0.01 in the GFI, TLI and CFI goodness-of-fit indices [73] and the *Δ* ≤ 0.015 in the RMSEA. In addition, we calculated the configural, metric, scalar, and strict scale invariance between Brazil and Spain. These calculations were also carried out with the JASP 0.16.3 software.

Finally, the reliability of the test of Brazil scale was studied through Cronbach's alpha and McDonald's Omega reliability indices using the JASP 0.16.3 software. The value of McDonald's Omega index is preferably considered, as the limitation of Cronbach's alpha derived from its sensitivity to changes in sample sizes has been extensively studied [74]. Since we were dealing with a large sample, with a total of more than 500 cases for this analysis, the sensitivity must be corrected. Therefore, similar values were observed for both reliability indices.

For the study of criterion validity, the procedure for the validation of the same test in the Spanish context was followed [54]. A correlational analysis was developed, first, for the total score of the JIS, and its dimensions, with the total mental health score of the GHQ, as well as with its subscales in the Brazilian version: somatic symptoms and anxiety; social dysfunction, and depression. Also with the Job Satisfaction score.

#### Study 2: Cross-national comparison: Brazil and Spain

Once the test was validated for use in the Brazilian context, a cross-cultural comparison of the effects of job insecurity between the samples of Spanish and Brazilian workers was carried out. For the comparison of the phenomenon, we compared the effect size of job insecurity in its affective and cognitive dimensions (IV) with respect to total mental health scores with samples from both countries (DV). This analysis was developed with a stepwise linear regression calculated with JASP 0.16.3, entering in sequence the cognitive and affective dimensions of the JIS (CI = 95%). Comparison of effect sizes is an appropriate procedure in this type of study [75], and as an index of effect size, following previous work [76], we used the explained variance which expresses the adjusted *R*^*2*^ for each independent variable in the model. This analysis was carried out with the scale validated in Brazil and the original Spanish scale with 8 items [54].

## Results

### Sample’s descriptive characteristics

The sample consisted of 1165 people, as indicated in the method section. In the sample as a whole, 38.2% were women and 61.8% men. 48.50% of the participants have a temporary contract and 51.5% have a permanent contract (Table 1) The mean age of the sample was 32.78 years for the full sample, being 28.71 years in the Brazilian part of the sample and 36.68 in the Spanish sample. Exploring each of the countries we find a similar balance between the different conditions exposed. In the Brazilian part of the sample (n = 573), 45.20% (n = 259) were men and 54.80% were women (n = 314). Some 64.22% had permanent employment (n = 368) and 35.78% had temporary employment (n = 205). Analyzing the Spanish part of the sample (n = 592), 31.42% (n = 186) were men and 68% (n = 406) were women. According to their employment status, 31.19% (n = 232) had a permanent job and 60.81% (n = 360) had a temporary job. However, chi-square (*χ2*) analysis for gender (*χ2* = 22.84, *p* < 0.01) and employment status between the two countries (*χ2* = 71.9; *p* < 0.01), as well as the t-student test for age (t = 13.19, *p* < 0.01), showed statistically significant differences between the groups. This implies taking with caution the conclusions of a comparative analysis between the countries, since there is no absolute equivalence in the characteristics of both samples.

**Table 1:** Sample description

		Age	Contract type	
	n (%)	Mean (SD)	Temporary workers (%)	Permanent workers (%)
*Brazil and Spain*				
Men	445 (38.20%)	32.36 (11.63)	191 (33.81%)	254 (42.33%)
Women	720 (61.80%)	33.04 (10.73)	374 (66.19%)	346 (57.66%)
Total	1165	32.78 (11.08)	565 (48.50%)	600 (51.50%)
Sample comparison between countries	*χ2* = 22.84**	t = 13.19**	*χ2* = 71.9**	
*Only Brazil*				
Men	259 (45.20%)	29.07 (8.85)	90 (43.90%)	169 (45.92%)
Women	314 (54.80%)	28.4 (8.81)	115 (56.10%)	199 (54.08%)
Total	573 (49.18%)	28.71 (8.83)	205 (35.78%)	368 (64.22%)
*Only Spain*				
Men	186 (31.42%)	36.87 (13.38)	101 (28.06%)	85 (36.64%)
Women	406 (68.58%)	36.59 (10.72)	259 (71.94%)	147 (63.36%)
Total	592 (50.82%)	36.68 (11.61)	360 (60.81%)	232 (31.19%)

**(*p* < .01)

### Study 1: Job Insecurity Scale (JIS-8), adaptation to Brazilian context

#### Job Insecurity Scale structure and invariance analysis

The exploratory factorial pre-analysis with the 8 items of the JIS scale in Brazil showed a good KMO (0.768). However, item number 4 (There is only a small chance that I will become unemployed) showed anomalous behaviour. In the Measure of Sampling Adequacy (MSA) test, this item showed a value close to 0.50, in contrast to the rest of the items with values close to and above 0.70. This indicated that the item was measuring an asynchronous domain with respect to the others, which reaffirmed a low communality (0.37). The factor loading of this item was poorly too (0.35 in his most relevant factor). Based on this evidence, the decision was taken to eliminate this item from the test. In the Spanish version of the scale, item 4 was maintained. However, its factorial weight was low (0.40) and substantially lower than the rest of the items.

The test is subjected to EFA with 7 items with the fourth item removed. The KMO index in this case was 0.77. The Optimal implementation of parallel analysis recommends the extraction of two factors, with the percentage of variance explained by the two factors in the real data being higher than that explained by the Randomly Generated data of random % of variance (Table 2). The MIREAL test (Mean of Item Residual Absolute Loadings) showed a value of 0.44, which made it possible to rule out with certainty that this was a one-dimensional test. A factorialisation into two dimensions coincided with the proposal of the scale in its original conceptualisation [52] and its validation in Spain [54]

**Table 2:** Optimal implementation of Parallel Analysis: Percentage of variance of real and randomly generated data

	Real-data % of variance	Randomly generated data of random % of variance
1	56.45**	29.18
2	27.67*	23.76
3	6.63	18.95
4	4.59	14.01
5	3.84	9.39
6	0.77	4.72

*p < .05

**p < .01

The Root Mean Square of Residuals (RMSR) was 0.027 with a good model fit [77]. The same approximation was observed in Weighted Root Mean Square Residual (WRMR), with a value below 1.0 (WRMR = 0.025 for our data) [78]. None of the items had a communality of less than 0.50, and the table of inter-item polychoric correlations shows relationships with significance (Table 3). The factor loadings with the Promin rotation method maintained the dimensional logic of the test in the Spanish population: a first factor (cognitive) with items 1, 2 and 3; and the second factor (affective) with items 5, 6, 7 and 8. The criterion for considering a factor loading to be relevant was set at 0.30 [66]. The test loadings ranged from minimum and maximum values of 0.66 to over 0.90 (Table 4).

**Table 3:** Polychoric correlations between items

Items	1	2	3	4	5	6
1. Tenho muita ateria de que serei capaz de manter meu emprego *(I am very sure that I will be able to keep my job)*	1.00					
2. Sinto-me ateri(a) no meu ambiente de trabalho *(I am certain/sure of my job environment)*	0.66*	1.00				
3. Penso que serei capaz de continuar trabalhando aqui *(I think that I will be able to continue working here)*	0.65*	0.70*	1.00			
5. Tenho medo de ser despedido(a) *(I fear that I might get fi red)*	0.27*	0.24*	0.06	1.00		
6. Estou preocupado(a) com a continuidade da minha carreira *(I worry about the continuation of my career)*	0.15*	0.23*	0.14*	0.56*	1.00	
7. Tenho medo de que possa perder meu emprego *(I fear that I might lose my job)*	0.26*	0.30*	.12*	0.85*	0.59*	1.00
8. Sinto incerteza sobre o ateri do meu emprego *(I feel uncertain about the future of my job)*	0.41*	0.46*	0.38*	0.58*	0.55*	0.70*

*p < .05

**Table 4:** Job Insecurity Scale (JIS) item values

Item	M	SD	Skweness	Kurtosis	r^a^	ω^b^	Factor loadings		Communality
							1	2	
1	2.53	1.06	0.43	− 0.09	0.53	0.81	0.10	**0.73**	0.71
2	2.65	1.07	0.36	− 0.42	0.55	0.81	0.11	**0.79**	0.73
3	2.53	1.05	0.50	− 0.17	0.45	0.82	− 0.10	**0.90**	0.74
5	2.87	1.29	0.13	− 0.96	0.58	0.79	**0.91**	− 0.15	0.91
6	3.27	1.28	− 0.34	− 0.88	0.45	0.81	**0.66**	− 0.04	0.52
7	2.91	1.25	0.09	− 0.83	0.66	0.77	**0.99**	− 0.13	0.91
8	3.00	1.16	− 0.08	− 0.65	0.65	0.78	**0.68**	0.23	0.76

Bolded data in the factor loadings indicate the factor to which the highest loading corresponds

r^a^ correlation item-rest; ω^b^ McDonald’s Omega if item dropped

**Fig. 1:**
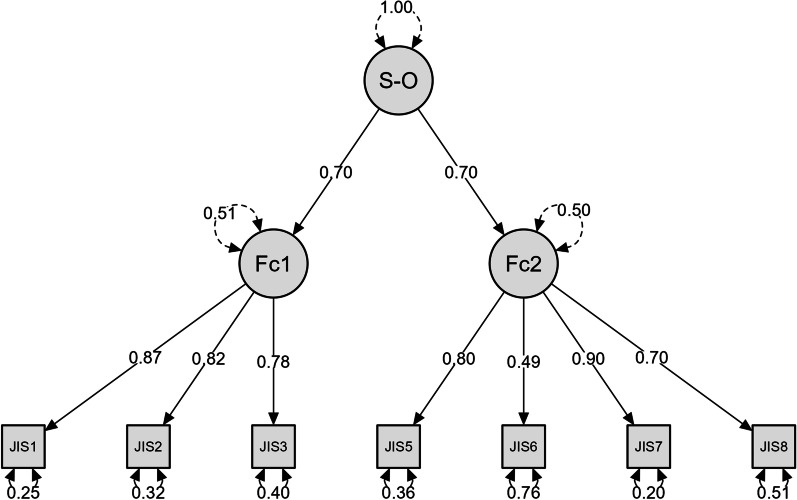
CFA Two factor model with a second order latent factor

The confirmatory factor analysis (CFA) tested the fit of the one-factor model, the fit of the model with two dimensions, and the fit of two dimensions with a second-order latent factor. The two-factor models fitted better than the one-factor model, and the one-factor model with a method factor. Table 5 shows that the one-dimensional model had a poor fit. On the other hand, the two-factor model and two factor model with a second-order latent factor had a perfect fit (Table 5) according to the fit criteria [68, 70]. In this case, the fit indices were almost identical. However, the two-factor model with a latent factor offered a global measure of the job insecurity construct, and this procedure replicated the Spanish validation of the scale, so it was convenient to choose the two-factor with a second-order latent factor model for the scale. Having a second-order latent factor allows us to analyse the scores of each subscale of the test, as well as to extract a total score (Fig. 1).

**Table 5:** Goodness-of-Fit Indices for the JIS in Brazil, and metric invariance of multigroup comparisons

CFA	CFI	TLI	RMSEA (CI 95%)	SRMR	GFI	NFI
Criterial values	≥ 0.95	≥ 0.95	≤ 0.10	≤ 0.08	≥ 0.95	≥ 0.95
One-Factor model	0.891	836	0.162 (0.134; 0.190)	0.123	0.992	0.878
Two-Factor model	0.994	0.990	0.04 (0.000; 0.078)	0.049	0.999	0.980
Two-Factor with second-order latent factor	0.993	0.987	0.04 (0.000;0.084)	0.049	0.999	0.980

Invariance test	X^2^	df	CFI	TLI	GFI	RMSEA	ΔCFI	ΔTLI	ΔGFI	ΔRMSEA
Base	913.274	21	0.993	0.987	0.999	0.045				
Configural	941.535	42	0.988	0.978	0.985	0.059	0.005	0.009	0.014	0.014
Metric	941.535	42	0.993	0.990	0.985	0.040	0.005	0.012	0.00	0.019
Scalar	941.535	42	0.996	0.995	0.997	0.028	0.003	0.005	0.012	0.012
Strict	941.535	42	0.998	0.998	0.997	0.016	0.002	0.003	0.00	0.016

The results of the multigroup Invariance of the factor structure showed configural, metric, scalar and strict invariance between men and women. The goodness-of-fit indices in all cases indicated an increase of less than or close to Δ ≤ 0.10. For the RMSEA the criterion was Δ ≤ 0.15. The metric invariance showed a small deviation, but in the rest of the cases the assumption was fulfilled (Table 5). Therefore, the structure of the scale did not vary between men and women.

When testing the invariance between countries, it is worth considering that the scale in Brazil has 7 items (item 4 is eliminated) while in Spain it has 8 items. In order to make the cross-country calculation possible, the Spanish scale was subjected to a CFA and reliability analysis with the sample, eliminating item 4. The structure was proposed in the Spanish data in the same way as in the Brazilian sample: one factor with items 1, 2 and 3, and another with items 5, 6, 7 and 8. In addition, a second-order latent factor was extracted. The model fit was adequate to the criterion: CFI = 0.998; TLI = 0.996; RMSEA = 0.027 (CI 95%: 0.006; 0.045); SRMR = 0.027; GFI = 0.998 and NFI = 0.996. Internal consistency was also adequate: McDonald’s ω for ordinal scales is ω = 0.90 for the full scale; ω = 0.84 for the cognitive dimension and ω = 0.86 for the affective dimension ω = 0.90 [82]. With these results we calculated the invariance for the scale with the sample of the two countries replicating the criteria of the invariance between men and women in Brazil. The goodness-of-fit indices in all cases indicated an increase of less than or close to Δ ≤ 0.10. For the RMSA the criterion was Δ ≤ 0.15, and the variation was smaller (Table 6). Therefore, the structure of the scale did not vary between countries (Brazil and Spain).

**Table 6:** Goodness-of-Fit Indices for the JIS in Brazil and Spain with 7 items, and metric invariance of multigroup comparisons

Invariance test	X^2^	df	CFI	TLI	GFI	RMSEA	ΔCFI	ΔTLI	ΔGFI	ΔRMSEA
Base	4883.168	21	0.995	0.991	0.997	0.041				
Configural	5227.581	42	0.993	0.988	0.994	0.051	0.002	0.003	0.003	0.010
Metric	5227.581	42	0.987	0.981	0.991	0.063	0.006	0.007	0.003	0.012
Scalar	5227.581	42	0.981	0.977	0.998	0.070	0.006	0.004	0.007	0.003
Strict	5227.581	42	0.977	0.976	0.997	0.071	0.004	0.003	0.001	0.001

#### Reliability and Validity

The internal consistency of the test measured with McDonald’s ω for ordinal scales awas ω = 0.82 for the full scale;* ω* = 0.84 for the cognitive dimension and ω = 0.84 for the affective dimension. These indicators pointed to a very good reliability for the scale and its dimensions [79]. Cronbach’s α data was very similar. The intraclass correlation is ICC = 0.358, and the Item reliability if item dropped showed little relevant variation (Table 4).

The JIS scale and its dimensions were correlated with mental health and job satisfaction scores, yielding significant correlations in all cases (Table 7). This replicated the successful validation procedure of the JIS test in the Spanish working population. Higher correlations were observed with the total job insecurity score and the cognitive dimension than with the affective dimension of the construct.

**Table 7:** Correlations between Job Insecurity Scale and its dimensions and other related variables

Variable	1	2	3	4	5	6	7
1. JIS Total	1						
2. JIS Affective dimension	0.089**	1					
3. JIS Cognitive dimension	0.74**	0.34**	1				
4. GHQ 28	0.36**	0.26**	0.34**	1			
5. GHQ Somatic Symptoms and Anxiety	0.31**	0.25**	0.27**	0.93**	1		
6. GHQ Social dysfunction	0.28**	0.16**	0.34**	0.72**	0.52**	1	
7. GHQ Depression	0.29**	0.21**	0.28**	0.76**	0.54**	0.50**	1
8. Job Satisfaction	0.21**	0.09**	0.30**	0.28**	0.24**	0.29**	0.20**

**p < .01

#### Study 2: Cross-national comparison: Brazil and Spain

The second objective of the study investigated the comparison between Brazil and Spain regarding the relationship between the cognitive and affective dimensions of the JIS (IV) scale with the total mental health score (GHQ-28) (DV) (Table 8). The regression model replicated with the samples from both countries was significant in both cases (*p* < 0.01): in the Brazilian sample the cognitive dimension presented a higher standardised beta (βstd = 1.35, *p* < 0.001) than the affective dimension (βstd = 0.54, *p* < 0.001). In Spain, the cognitive dimension (βstd = 0.47, *p* < 0.001) and the affective dimension (βstd = 0.43, *p* < 0.01) presented similar beta results.

**Table 8:** Linear regression

	B (CI 95%)	Standard error	B standarised	t	Sig	R^2^ change	R^2^ Adj. change
*Brazil*							
(constant)	7.52	1.91					
JIS Cognitive	1.35 (0.954; 1.743)	0.20	0.29	6.72	< 0.001	0.118	0.116
JIS Affective	0.54 (0.262;0.808)	0.14	0.16	3.85	< 0.001	0.024	0.022
*Spain*							
(constant)	12.44	1.70					
JIS Cognitive	0.43 (0.094; 0.774)	0.17	0.12	2.5	0.01	0.01	0.01
JIS Affective	0.47 (0.208; 0.739)	0.14	0.17	3.5	< 0.001	0.059	0.06

Comparative Brazilian and Spanish workers. GHQ total as dependent variable

When comparing effect sizes, the R^2^ Adj in the Brazilian case (R^2^ Adj = 0.138) was larger than in the Spanish sample (R^2^ Adj = 0.07). It was observed that job insecurity had a much greater explanatory capacity for variability in mental health in the case of the Brazilian sample. Analysing the independent variables separately, in the Brazilian case there was a substantial difference between the variability explained by affective (R^2^ Adj = 0.022) and cognitive (R^2^ Adj = 0.116) job insecurity; while in the Spanish sample the data showed an inverse relationship: affective job insecurity (R^2^ Adj = 0.06) had a greater explanatory capacity than cognitive job insecurity (R^2^ Adj = 0.01). It should also be noted that the two job insecurity variables were included in the model for both countries.

## Discussion

### Study 1: Job Insecurity Scale (JIS-8), adaptation to Brazilian context

The first objective of this study was to validate the JIS scale [52] in the context of Brazilian employees. This objective was achieved by having a scale for measuring subjective job insecurity with high reliability indices and a factor structure that coincides with the baseline test [52], as well as the one resulting from the Spanish validation [54]. The invariance between countries has also been tested, which implies correspondence of the scale between both contexts. The Spanish adaptation study pointed out the relevance of developing psychometric scales for the measurement of the phenomenon in Latin America, and more specifically in the Brazilian case where there is no precedent. It is also the first adaptation of this scale to use polychoric correlations in its confirmatory factor analysis. This method is not only more suitable for Likert-5 items, but the proposed factor extraction is more conservative with respect to multidimensionality than the principal component analysis usually used in this type of design [66]. Despite this, the test has been adjusted to a two-factor model with a latent-one, with the implications that will be discussed below.

The scale in the Brazilian validation presents one less item, with the elimination of the fourth Item. This item, as mentioned above, had substantially lower factor weights than the rest in Spain and in the original validation, which is considered a congruent decision. One of the factors, with this elimination, is now measured in three items, which is considered acceptable for the measurement of a dimension in a psychometric instrument [80]. The reverse direction of four of the items is maintained, in order to seek fidelity with the original scale and its different versions.

The validity study of the instrument reveals significant correlations between scores in perceived job insecurity and measurements of mental health and job satisfaction. In the case of job satisfaction, an inverse correlation is found with all measurements of job insecurity in the scale, and a direct correlation with mental health. These results are consistent with the scientific literature [51, 54]. This supports not only that the scores are adequate to establish criterion validity, but also the correct behaviour of the scale in its cross-cultural adaptation to the Brazilian population. It should be noted that the correlation between the total job insecurity score and the mental health variable is higher than that observed with job satisfaction. This is in continuity with the work of Stiglbauer et al. [81] where a higher correlation of job insecurity with cognitive well-being (r = − 0.35***) than with variables related to job involvement (r = − 0.12) is observed. It also shows a high relationship between job insecurity and intention to leave (r = 0.48***). This leads to the conclusion that job insecurity is a high intensity phenomenon, which is more related to the negative impact consequences of its occurrence. At the same time, it reflects the fact that in order to understand the effects of job insecurity, it is not only necessary to observe labour-related variables, but it also involves the person’s state of well-being in its broadest sense. That is to say, the expectation of job loss conditions the person’s situation in the rest of the spheres of life development. This is based on theoretical approaches that understand precarious employment as precarious living [82]: precarious employment—in this case the dimension of job insecurity is measured—cannot be isolated from the subject’s quality of life. Låstad et al. [83] also relates quantitative perceived job insecurity more strongly to the deterioration of psychological well-being (r = − 0.26***), than to other work-related variables such as work/family conflict (r = 0.18***).

In the above sense, the relationship between job insecurity and other variables, such as health, shows that job insecurity is a measure of job precariousness [15]. The conceptualisation of job insecurity is not only broad, but necessarily mutable. In this sense, we provide new evidence on the relevance of looking at job insecurity as an indicator of job insecurity, in line with other authors [84]. In fact, in line with the flexible and volatile nature of the labour market [14], definitions of job precariousness that focus solely on objective aspects do not take into account the magnitude of the phenomenon under study. Thus, proposals for conceptualising precariousness such as that by Vosko [85], whereby “precarious work can be defined as employment characterised by insecurity, low pay and limited social benefits” [p. 2], reflect how job insecurity is an intrinsic element for its understanding, through a measurement of the subjective experience of job insecurity.

Another aspect to discuss is the factorialisation of the test into two dimensions. This involves discussing the multiple conceptual models of subjective job insecurity. In this sense, the validation of the instrument in Brazil opted to explore a second-order latent factor in order to give the instrument versatility. The adjustment of the two-factor model, cognitive and affective, together with the total score extracted with the second-order latent factor, allows three scores to be extracted from the test. This provides an answer to those authors who see perceived job insecurity as a one-dimensional construct [19], as it ateri a total score to be obtained through the scale in Brazil. However, the results of the validation process are clear in defining a two-dimensional model (affective and cognitive). In this sense, it has been shown that the impact of affective job insecurity is differential, with higher correlations with the mental health and job satisfaction scales in the case of cognitive job insecurity. Sverke et al. [86] argued in their classic meta-analysis that affective job insecurity was what “best reflect(s) the conceptual definition of job insecurity” [p. 256] However, our research invites further work with the two-factor model. First, because the CFA shows this dimensionalisation to be feasible. Also, because the regression analysis conducted on the Brazilian population shows that cognitive job insecurity has a greater explanatory capacity on workers’ mental health. In this sense, most of the existing scientific literature on job insecurity comes from the Western social and labour context [20], which means that bringing the concept closer to employment frameworks such as that of Latin America contributes new evidence for its conceptual exploration.

### Study 2: Cross-national comparison: Brazil and Spain

A recent meta-analysis showed that affective job insecurity tends to be more important than cognitive job insecurity in mental health [48]. Looking at the regression study designed with the Brazilian sample and general mental health as the dependent variable, the results reflect, however, a more significant *R*^*2*^ for cognitive job insecurity (R^2^ adj = 0.116) than for affective job insecurity (R^2^ adj = 0.022). Both dimensions of job insecurity are significant in the linear regression model. Recent literature reports such behaviour when studying the phenomenon in a statistically representative sample of subjects, in which cognitive job insecurity has a greater influence on mental health than affective job insecurity [86]. If we refer to the original validation of the instrument being validated, it is also evident that cognitive job insecurity explains more variance in the mental health measure (βstd = 0.39) than affective job insecurity (βstd = 0.05) [52]. Salas-Nicas et al. [86] argue that it is difficult to explain the differential behaviour of the two factors at the moment. Evidence of this complexity is found in the data analysed with the Spanish sample of the study, where affective job insecurity has a greater weight in the explained variance of general mental health. The direction of the Spanish data is congruent with the aforementioned meta-analysis [48]. However, it should be borne in mind that the vast majority of analyses of this phenomenon have been carried out in the European context [40, 40, 43, 87, 88]. In contrast, our results show that the phenomenon presents different levels of intensity according to the context in which it is studied, meaning that cross-cultural comparative approaches, with a psychosocial perspective, are necessary. This had already been identified as a priority and pending task in the analysis of job insecurity [20].

Cross-national comparison exercises have been little explored with Latin American countries. Having the scale adapted to the Brazilian context has allowed us to compare a sample of Spanish and Brazilian populations, which was the second objective of the study. When comparing the effect size of perceived job insecurity on mental health measured with the GHQ, the effect sizes among the Brazilian working population (R^2^ Adj = 0.138) are substantially higher than those obtained in the Spanish sample. They are higher in all cases, both for the affective dimension and the cognitive dimension. The percentage of variance explained in the case of the model developed in Spain (R^2^ Adj = 0.07) is very similar to that seen in previous studies with the same variables (R^2^ Adj = 0.06) [54] It can be concluded, therefore, that job insecurity is more relevant for understanding the mental health of workers in the Brazilian context than in a European context such as Spain.

Interpreting these results requires analysing the contrast between the social contexts of the two countries. These data require an interpretation of the recent labour history of both territories. Both the Spanish and Brazilian cases go through authoritarian periods in the second half of the twentieth century, which implies that the construction of their social and labour protection models are late and fragile [7, 89]. In the Spanish case, this aterialized in the duality of labour at the turn of the millennium. In the Brazilian case, it is the central-peripheral inequality of conditions (tertiary labour framework of the big cities versus the agrarian labour framework of the periphery), as well as the structural integration of informal work. However, both countries are moving in unison towards the deregulated social and labour realities of neoliberal contexts. In this sense, a clear differentiation appears: in the Spanish case, the labour market continues to be fragile, but within the European Union’s economy [90]. This implies a stronger economy than in Brazil, with a social protection system that provides more guarantees for employees. In the Brazilian case, there is a tendency towards economic subsidiarity with European countries and North America, which, together with the prior fragility of employment, places the Brazilian labour context in a situation of poorer protection [7]. Against this background, the scientific literature has shown that job insecurity is a phenomenon mediated by conditions of poverty and resource availability [15, 91]. Thus, those who live with fewer resources experience greater job insecurity and its effects more intensely: both because the jobs they tend to have access to are more precarious, and because the cost of losing a job is higher. Studies on job insecurity around periods of economic crisis also reflect this situation of increased value of work due to the present economic instability [16, 24]. At the same time, the perception of social inequality has been found to have a moderating effect on the consequences of job insecurity [92]. If we compare inequality indicators in the Brazilian macro socio-economic context, the GINI index exceeds the Spanish one by 19.2 points according to World Bank data [60]. Regarding the employment situation in the Brazilian and Spanish contexts, we do not see very different trajectories in unemployment rates. However, the vulnerable employment rate as formulated by the ILO [93] shows that in Brazil it is 17 percentage points higher than the already high Spanish rate (11% in Spain, compared to 5% in Germany and 8% in France) [58]. Once again, we are forced to link the concept of job insecurity with expectations [94, 95], and the expectation of job development in a fragile social and occupational market can be hypothesised to influence the experience and consequences of job insecurity.

### Limitations and future research

As for the limitations of this research, it would be necessary to go deeper with more data to explain why there is this differential influence between the impact of job insecurity in Spain and Brazil. In this sense, the availability of this validated psychometric scale opens up the possibility of analysing the phenomenon in the territory, which until now was not possible in these terms. Secondly, the discussion remains open regarding the differential relationship between the affective and cognitive dimensions of mental health. In this sense, a deeper understanding of the social conditions of the contexts can help us to observe the conditioning factors that intervene in the phenomenon [86]. Thirdly, although the test has been translated into Portuguese, it has only been adaptated in the Brazilian context, so it would be necessary to develop a cross-cultural adaptation process in Portugal for its use in this context. The paper also reflects some methodological limitations, such as its cross-sectional design. Although it is a common method for psychometric adaptation studies, it would have been desirable to design a repeated measures approach that enabled the calculation of the intra-class correlation coefficient to strengthen the evidence of internal consistency.

Regarding the characteristics of the sample, data on ethnic characteristics are not available for the Brazilian sample, which would be an important aspect to address in future work on job insecurity in the Brazilian context. Finally, the analyses of the sample between the two countries show that there is no absolute equivalence in the characteristics of both samples (Spanish and Brazilian) in their socio-labor conditions. This may condition the results of the study, so caution is required when making generalizations about the conclusions of this work.

## Conclusion

In conclusion, the Brazilian Job Insecurity Scale (JIS) adapted in this paper offers an instrument with psychometric guarantees. It maintains the factorial structure in two dimensions, cognitive and affective, with an extension of 7 items. Therefore, three scores can be extracted: a total score on job insecurity, as well as a score on affective job insecurity and another on cognitive job insecurity.

The JIS scale also offers the possibility of performing cross-national analysis. This study compares the phenomenon with the Spanish working population, observing a differential relationship between job insecurity and mental health in both countries. Mental health in Brazil is most compromised by experiencing job insecurity among employed people. The effects of labor insecurity in Spain are like those in the rest of Europe. These results justify the relevance of establishing an analysis of the labor reality in Latin America through the study of Job Insecurity.

## Data Availability

The dataset is available from the corresponding author on reasonable request.

## Abbreviations

ILO: International Labor Organization
QUAL-JIS: Qualitative Job Insecurity Scale
JIS: Job Insecurity Scale
OCED: Organisation for Economic Co-operation and Development
GHQ-28: Goldberg General Health Questionnaire
EU LFS: European Union Labour Force Survey
WMA: World Medical Association
ULS: Unweighted Least Squares
EFA: Exploratory Factor Analysis
CFA: Confirmatory Factor Analysis
CFI: Comparative Fit Index
TLI: Tucker-Lewis index
RMSEA: Root mean squared error of approximation
GFI: Gaussian Fuzzy-index
NFI: Normed Fit Index
IV: Independent variable
DV: Dependent variable
KMO: Kaiser–Meyer–Olkin measure
CI: Confidence Interval
MSA: Measure of Sampling Adequacy
MIREAL: Mean of Item Residual Absolute Loadings
RMSR: Root Mean Square of Residuals
WRMR: Weighted Root Mean Square Residuals
ICC: Intraclass Correlation
